# Future complications of chronic hepatitis C in a low-risk area: projections from the hepatitis c study in Northern Norway

**DOI:** 10.1186/s12879-017-2722-0

**Published:** 2017-09-16

**Authors:** H. Kileng, L. Bernfort, T. Gutteberg, O.S. Moen, M.G. Kristiansen, E.J. Paulssen, L.K. Berg, J. Florholmen, R. Goll

**Affiliations:** 1 Gastroenterology and Nutrition Research Group, Department of Clinical Medicine, UiT The Arctic University of Norway, Tromsø, Norway; 2 Department of Medical and Health Sciences, University of Linköping, Linköping, Sweden; 3 Department of Internal Medicine, Section of Gastroenterology, University Hospital of North Norway, Tromsø, Norway; 4 Research Group for Host-Microbe Interactions, Department of Medical Biology, UiT The Arctic University of Norway, Tromsø, Norway; 5 Department of Microbiology, University Hospital of North Norway, Tromsø, Norway; 6 Department of Medicine, Nordland Hospital, Bodø, Norway; 7 Department of Medicine, Helgeland Hospital, Mo i Rana, Norway

**Keywords:** Disease burden, Fibrosis development, Hepatitis C, Markov modelling, Natural course

## Abstract

**Background:**

Hepatitis C (HCV) infection causes an asymptomatic chronic hepatitis in most affected individuals, which often remains undetected until cirrhosis and cirrhosis-related complications occur. Screening of high-risk subjects in Northern Norway has revealed a relatively low prevalence in the general population (0.24%). Despite this, late complications of HCV infection are increasing. Our object was to estimate the future prevalence and complications of chronic HCV infection in the period 2013–2050 in a low-risk area.

**Methods:**

We have entered available data into a prognostic Markov model to project future complications to HCV infection.

**Results:**

The model extrapolates the prevalence in the present cohort of HCV-infected individuals, and assumes a stable low incidence in the projection period. We predict an almost three-fold increase in the incidence of cirrhosis (68 per 100,000), of decompensated cirrhosis (21 per 100,000) and of hepatocellular carcinoma (4 per 100,000) by 2050, as well as a six-fold increase in the cumulated number of deaths from HCV-related liver disease (170 per 100,000 inhabitants). All estimates are made assuming an unchanged treatment coverage of approximately 15%. The estimated numbers can be reduced by approximately 50% for cirrhosis, and by approximately one third for the other endpoints if treatment coverage is raised to 50%.

**Conclusion:**

These projections from a low-prevalence area indicate a substantial rise in HCV-related morbidity and mortality in the coming years. The global HCV epidemic is of great concern and increased treatment coverage is necessary to reduce the burden of the disease.

**Electronic supplementary material:**

The online version of this article (10.1186/s12879-017-2722-0) contains supplementary material, which is available to authorized users.

## Background

Chronic hepatitis C virus (HCV) infection is a major cause of chronic liver disease and the burden of the disease is expected to increase [1–3]. Worldwide, 64–103 million people are persistently infected with HCV [4]. After acute HCV infection, between 75% and 85% of the patients establish a chronic infection [5]. In industrial countries, most of the patients infected with HCV will have contracted the disease in the 1970s and 1980s. Thus, at the beginning of the twenty-first century, a large pool of infected patients exists, and in many countries, the cohorts of patients with chronic HCV infection have come of age. Most infected individuals are asymptomatic, but the number of patients with liver cirrhosis and hepatocellular carcinoma (HCC) is increasing [6, 7]. Thus, 30–40 years after the start of the epidemic, the disease is a growing burden on the health care system in many countries.

The natural course of late-stage HCV infection is so far unsettled. The disease increases the risk of developing liver cirrhosis and its complications such as liver cancer and liver failure. The prognosis is highly dependent on the rate of progression of liver fibrosis towards cirrhosis. Most studies have shown that the rate of fibrosis is slow the first two decades. However, the estimated risk of cirrhosis varies as much as 3–30% in different populations [5, 8–10], suggesting that the progression of the disease may not be universal but rather depend on additional risk factors [11]. Beyond the two first decades, the rate of fibrosis progression is sparsely documented. One study reported that fibrosis progression is non-linear with an estimated a risk of cirrhosis of 41% after 30 years [8], whereas an autopsy study reported septal fibrosis or cirrhosis in 35% of cases with disease duration of 25 years or longer [12].

Among patients with liver cirrhosis the annual rate of progression to hepatic decompensation and HCC has been described to be in the range 4–8% and 2.4–3.4%, respectively [13–15]. In the absence of retrospective as well as prospective data for the long-term progression of the disease (>20 years), various mathematical models have been used to reconstruct the natural course and estimate future complications of HCV infection [1, 13, 16–18]. Even with a decline in the incidence rate after 1990, an increase in the number of patients with complicated disease and deaths from chronic HCV is expected in the coming decades [1–3, 19, 20].

Norway is a country with a low prevalence of HCV [21, 22]. Drug abuse is the primary transmission route, and low age at transmission indicates a low risk for rapid disease progression. Despite this, late complications of HCV infection is a growing problem [23] and health costs are expected to increase. Markov models are well suited for simulation of chronic diseases [24], and we have used a Markov model to estimate the future prevalence and complications of chronic HCV infection in the period 2013–2050 in a low-risk area.

## Methods

The study population consists of individuals included in the Hepatitis C study in Northern Norway between 1992 and 2011 [21, 23]. In addition, we performed a registration study to assess the number of newly diagnosed individuals with HCV each year between 1998 and 2012 at the two microbiological departments in our region.

### The Hepatitis C study in Northern Norway

In 1992, a screening and medical follow-up programme of patients with HCV infection was established in the Health Region of Northern Norway (460.000 inhabitants). In brief, general practitioners were encouraged to screen patients with former or present risk behavior for HCV infection. If chronic HCV infection was detected, the general practitioners were encouraged to refer the patient for follow-up at one of the 11 medical centers in the region. An estimate of the year of transmission was made for all referred patients based on either the year of acute HCV infection or the first year of high-risk behaviour [23]. Liver biopsies were performed, and fibrosis was graded (0–6) according to Ishak et al. [25]. Presence of concomitant alcoholic liver disease was assessed by clinical judgment.

### The registry study

Individuals with a positive anti-HCV test registered at the two microbiological departments in Northern Norway between 1998 and 2012 were included. The year of diagnosis was defined as the first year of a positive anti-HCV test (ARCHITECT Anti-HCV Reagens kit. Abbott System, Wiesbaden, Germany). Until 2004, a positive anti-HCV test was directly confirmed with a recombinant immunoblot assay (RIBA HCV 3.0 SIA test, Chiron Cooperation, Emeryville, CA, USA). Individuals with a positive RIBA (two or more positive bands) or indeterminate RIBA (one band) were included, while individuals with a negative RIBA (no bands) were excluded. The result of the HCV RNA test was recorded if available: an in-house reverse transcriptase polymerase chain reaction (RT-PCR) until 2004, where after the ROCHE RT-PCR (Cobas Amplicor Hepatitis C Viral Polymerase Chain Reaction, Roche Molecular System Inc., Branchburg NJ, USA) was used. The ROCHE PCR test replaced the RIBA test for confirmation of HCV infection from 2005. HCV genotyping was performed as a hybridization assay on products from the HCV RNA PCR according to the manufacturers’ instructions (INNO-LIPA HCV II kit, INNOGENETICS, Ghent, Belgium). Individuals without a registered residence in Norway were excluded. The results of HBsAg (hepatitis B surface antigen) and anti-HIV (human immunodeficiency virus) were recorded if available.

### Markov model

**Fig. 1:**
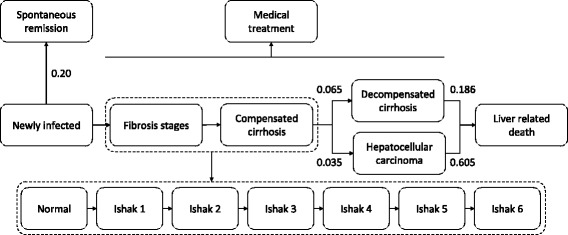
The natural course of hepatitis C. The Markov model is based on simulation of transition from a given category to the next based on probability estimates. The progression of fibrosis towards cirrhosis is based on local data, whereas most of the remaining estimated probabilities are previously published from other cohorts. In all states of HCV, there is also an annual risk of dying from natural courses, which is implicitly included in the model

In order to predict HCV-related morbidity and mortality an open Markov model was constructed (Fig. 1). Of infected patients, 20% were assumed to recover spontaneously during the first year of infection [26]. Yearly transitions between categories were done according to probability estimates generated from different sources. The effect of medical treatment was modelled in three scenarios where 0%, 15% and 50% of all patients were assumed to receive medical treatment. Before the introduction of direct acting antivirals (DAAs), treatment was offered to eligible patients irrespective of fibrosis stage, i.e. patients with Metavir fibrosis score F0/F1 were also treated. The treatment coverage at this time was approximately 15%. When the highly expensive DAAs became available, the Norwegian national guidelines restricted treatment to patients with fibrosis score ≥ F2. However, since DAAs offer a simple, tolerable, short-term and highly effective therapy, we consider an increased treatment coverage to 50% as possible. Recently (March 2017), updated national guidelines recommend treatment for all patients with genotype 1, irrespective of fibrosis grade. Treatment of genotype 2 and 3 is still restricted to patients with fibrosis score ≥ F2.

To estimate the rate of fibrosis progression in the cohort, 237 records with a positive HCV RNA test, known duration of infection, and available liver biopsy results were entered into an ordinal regression analysis, which can be described as a multilayer logistic regression. This method estimates points of transition on an ordinal scale (like the Ishak fibrosis scale). Sex, time after contraction of the disease and HCV genotype were included as predictors of Ishak fibrosis grade. Non-significant terms were removed from the model leaving time after contraction and genotype as significant predictors. Genotype 1 and 4 were analyzed as a single group due to few observations of genotype 4. The model predicted 70% of the observed fibrosis grades correctly within a margin of error of one Ishak grade, but overestimated fibrosis grade in 13% and underestimated in 17%. From this model, a matrix of estimated probabilities of transition to a higher fibrosis grade versus staying in the present grade for each year of infection could be constructed. It was assumed that fibrosis development during ongoing HCV infection could only either stay the same or change to a higher stage each year of infection. Additional file 1 shows the regression model in more detail. The Markov model was adjusted for genotypes in the estimation of fibrosis progression, according to the prevalence of the different genotypes in our population. For transitions from Ishak 6 (compensated cirrhosis) to more severe states, fixed probabilities were used. We did not have exact data from Northern Norway for the various transitions, and have thus used data from a Scottish HCV population of drug-injecting abusers (as in our study), as described by Hutchinson et al. [13], with transition probabilities similar to that reported by others [14, 27], as shown in Table 1. The model was corrected for standardized mortality rate according to Norwegian population characteristics. Non-cirrhotic subjects successfully treated for HCV with achieved sustained virological response (SVR) were removed from the model. Subjects with cirrhosis remain in risk of liver complications in spite of SVR, and of those, 50% were retained in the model [28]. All successfully treated subjects with decompensated cirrhosis and HCC were kept in the model.

**Table 1:** Transition rates between different states of hepatitis C

Transition rates			
From state	To state	Yearly transition rate	Reference
Infection time	Spontaneous remission	0.200	Thomas Clin Liver Dis 2005 [26]
Compensated cirrhosis	Decompensated cirrhosis	0.065	Hutchinson Hepatol 2005 [13]
Decompensated cirrhosis	Liver-related death	0.186	Hutchinson Hepatol 2005 [13]
Cirrhosis	Hepatocellular carcinoma	0.035	Hutchinson Hepatol 2005 [13]
Hepatocellular carcinoma	Death	0.605	Hutchinson Hepatol 2005 [13]

The table shows the estimated rates of transition between different states of development of hepatitis C and its complications, as used in the Markov model

The overall SVR rate until 2015 was 71.3%, based on the response of interferon-based treatment in our own clinical practice. For individuals treated from 2015 onwards, the overall SVR rate was set to 90% to reflect the improved treatment response provided by DAAs.

### Entering of data into the Markov model

**Fig. 2:**
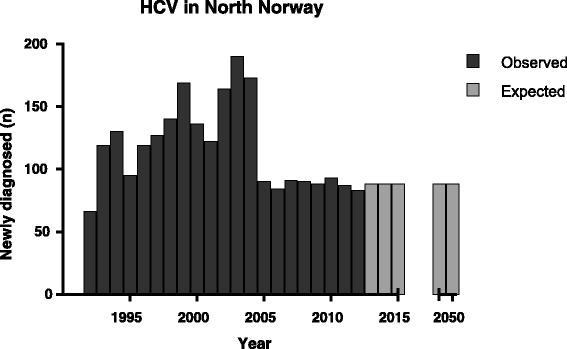
Newly diagnosed hepatitis C in Northern Norway. The figure shows numbers per year of newly diagnosed individuals with hepatitis C in Northern Norway, from the first registration in 1992 to the end of 2012 (dark bars). The estimated yearly occurrence of newly diagnosed from 2013 and forward are also shown (light bars)

Figure 2 shows the actual incidence of newly diagnosed HCV infection per year in the period from 1992 to 2012 (dark bars). After 2004, the incidence decreased to a stable level of approximately 90 newly diagnosed cases each year. In view of this stable number, we have projected a stable occurrence of 90 newly diagnosed cases each year from 2013 and forwards (light bars).

HCV-positive individuals were entered into the model at time of contraction. Since only a subgroup had a known time of transmission, this had to be estimated for the remaining records. First, we investigated the 402 records with known times of transmission. In 2004, all general practitioners in the area were subject to a HCV campaign encouraging screening of their patients with known risk behaviour. We found an increased amount of early diagnoses (within the first year of transmission) in the years after this campaign compared to before (35.5% vs. 21.3%). From these data, probability distributions could be derived in order to estimate time of contraction for records diagnosed before and after 2004, respectively. Records without known time of transmission were then entered into the model using these distributions as weights. From 2013 forward, the estimated number of 90 newly diagnosed cases each year were allocated due to the late probability distribution in order to estimate their time of contraction.

The registration of HCV infection revealed incomplete records regarding confirmation testing. A positive anti-HCV test normally should be followed up with a new blood sample for the confirmation with RIBA and/or HCV RNA. Not all the persons with positive anti-HCV tests had a recorded confirmation test. The starting cohort in the model thus consists of patients with confirmed HCV infection (either a positive RIBA or a positive HCV RNA test), as well as individuals with unconfirmed HCV (either only a positive anti-HCV or an indeterminate RIBA). We therefore estimated the likelihood of a true positive test in incomplete records in the following way: In a sample of 326 records with a positive anti-HCV-test where RIBA had been measured, 207 subjects (63%) had a positive RIBA test, and the probability of a true positive anti-HCV test was estimated to 0.63. Similarly, in a sample of 14 records with an inconclusive RIBA and a HCV RNA test, we found three individuals with a positive HCV RNA test, and the probability of a true positive record in case of inconclusive RIBA was estimated to 0.21. Summarized, individuals with either a positive HCV RNA or RIBA were weighted 1.0, and individuals with only a positive anti-HCV test or an inconclusive RIBA were weighted 0.63 and 0.21, respectively.

## Results

Based on the registration and subsequent weighting described above, the estimated HCV cohort consists of 2589 individuals (positive HCV RNA or positive RIBA) with a sex distribution of 64% men and 36% women. Additional file 2 shows the estimation of the HCV cohort in more detail. The distribution between genotypes 1 through 4 was 45%, 8%, 46% and 1%, respectively. The prevalence of HBsAg and anti-HIV was 2.3% and 1%, respectively.

### Modeled fibrosis progression

**Fig. 3:**
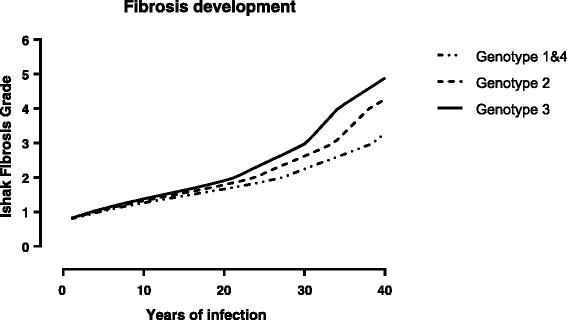
Development of fibrosis in hepatitis C. The figure shows the time course of distribution of estimated median fibrosis grade according to genotype and duration of HCV disease. Estimates were derived by ordinal regression analysis of Ishak scores in 237 patients with duration of infection and genotype as independent variables

In the subgroup that was the basis for estimation of fibrosis progression (*n* = 237), the sex distribution was the same as in the total cohort and the distribution between HCV genotypes 1 through 4 were approximately equal to the total cohort, 45%, 10%, 44%, and 1%, respectively. The median duration of infection was 13 years (range 0–42 years) and the mean age at liver biopsy was 40 years. The rate of fibrosis progression was relatively slow in the first 20–25 years of infection, followed by an accelerated fibrosis progression, especially in patients with genotype 3 (Fig. 3).

### Markov modelling

**Fig. 4:**
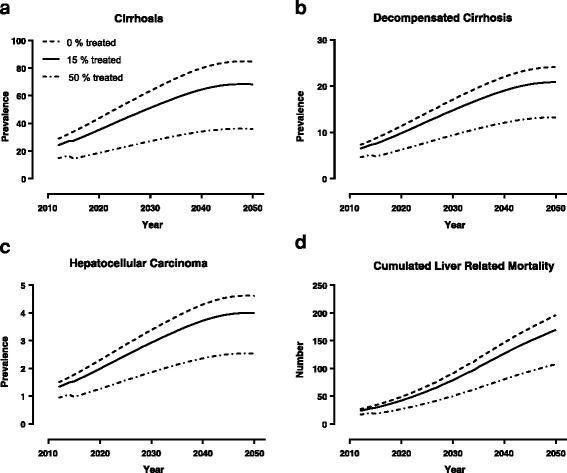
Prediction of hepatitis C-related complications. The figure shows the predicted number of patients with cirrhosis, decompensated cirrhosis and hepatocellular carcinoma (panels **a**-**c**), and predicted cumulated number of deaths from liver disease (panel **d**) in the period 2013 to 2050 according to different treatment coverage. Numbers are given per 100,000 inhabitants

The Markov model projects the number of patients (per 100,000 inhabitants) in the different states of compensated cirrhosis, decompensated cirrhosis and HCC for the years 2013 to 2050, given various scenarios of HCV treatment coverage. It estimates an almost threefold increase in the incidence of cirrhosis (68 per 100,000), of decompensated cirrhosis (21 per 100,000) and of hepatocellular carcinoma (4 per 100,000) by 2050, as shown in Fig. 4a-c. Complications are expected to reach a peak around 2040. The model predicts a six-fold increase in the cumulated number of deaths from HCV-related liver disease (170 per 100,000 inhabitants), as shown in Fig. 4d. All estimates are made assuming an unchanged treatment coverage of approximately 15%. The estimated numbers can be reduced by approximately 50% for cirrhosis, and by approximately one third for the other endpoints if treatment coverage were scaled up to 50%.

## Discussion

In our low-risk area of HCV infection, we have estimated the fibrosis progression in untreated hepatitis C. Fibrosis develops slowly the first 20–25 years of infection, where after an accelerated fibrosis progression, especially in genotype 3, is predicted.

Using a Markov model, we have estimated the future complications of HCV based on the actual number of infected individuals until the year 2012, followed by an estimated low and stable incidence rate. The model predicts a gradual increase in HCV-related liver cirrhosis, decompensated cirrhosis and HCC with an apparent peak around 2040, accompanied by a gradual increase in liver-related deaths.

### Modelling

Precision of dynamic modelling for prognosis of prevalence, morbidity and mortality depends on the quality of the data entered and the assumptions made. We have used locally acquired data whenever possible. The critical aspect in HCV pathophysiology is the progressive development of fibrosis towards cirrhosis, and in this aspect, our model also made use of local data. The further development from established cirrhosis is not likely to be very different from that of other cohorts, and the use of estimated transition rates from other studies [13] should not affect the prognosis measurably.

Our Markov model is based on an assumed low and stable incidence until 2050, whereas other reports estimate a declining or stable incidence [2]. The observed incidence peak in 2002–2004 in our data is probably a result of increased focus on HCV infection among general practitioners due to our encouragement of diagnosis in that period.

Our cohort is relatively young, and the liver biopsy data has a limited number of observations of severe fibrosis (Ishak grade 4–6). Hence, the estimates generated from these data are less precise regarding progression through high fibrosis stages.

Published data show variable rates of liver fibrosis progression, with 10%–40% developing cirrhosis after 20–35 years of infection [5, 8, 29]. A large systematic review supports nonlinear fibrosis progression [8] and other studies have shown that HCV genotype 3 is associated with a faster fibrosis progression [30–33]. Male sex is a reported risk factor for disease progression [5, 31]. However, we found no effect of sex in the progression of fibrosis, which is also observed in other studies [34].

#### Strengths and weaknesses

The strengths of this study are; first, we have reliable data on the HCV-infected subjects in our health region; second, the simulated fibrosis progression in the cohort is based on local data. However, there are apparent weaknesses. First, the number of undiagnosed HCV-infected individuals in the population is unknown. In Sweden, a comparable country regarding HCV epidemiology, it has been suggested that the rate of undiagnosed HCV infection is approximately 20% [35]. Second, incomplete records regarding confirmation testing has made it necessary to estimate the number of true positive cases, resulting in uncertainty regarding the true prevalence. However, our estimate of 63% true positives among those who only tested positive for anti-HCV is not controversial compared to another reported value of 68% true positive [36]. Moreover, the significance of RIBA-indeterminate reactions is unclear. Most individuals with indeterminate RIBA have a negative HCV RNA test, which may represent previous exposure to, but spontaneous recovery from HCV [37]. Authors have shown that approximately half of those with indeterminate RIBA have a resolved HCV infection [38, 39]. The proportion of RIBA-indeterminate records in our HCV population was low, making the contribution of these records less significant.

Third, the future incidence may be underestimated. Most likely, transmission of HCV will still be mainly by drug abuse, which is an ongoing problem in our region as is the case in the rest of Europe [40]. On the other hand, the introduction of highly effective, simple, short-term and tolerable therapies has the potential to increase treatment coverage among people who inject drugs. Thus, scaling up treatment in people with a transmission risk could reduce the future incidence and have a major impact on the HCV prevalence [41]. Fourth, it is documented that both moderate and excessive alcohol intake increase fibrosis progression in patients with HCV [42, 43]. Presence of concomitant excessive alcohol use was in our study assessed by clinical judgment, and not by a validated questionnaire of drinking habits. We have therefore chosen to omit alcohol use from the model over concerns on the quality of the data. Several other factors have been shown to accelerate the fibrosis progression, like co-infection with hepatitis B virus or HIV, diabetes and obesity [9]. The prevalence of HBV or HIV co-infections in our HCV population were low, 2.3% and 1% respectively, and therefore not included in the model. Data regarding diabetes and obesity were incomplete and neither included in the model.

Fifth, the exact time of infection is often not known. In individuals with unknown time of infection, we have used the first year of high-risk behavior as the presumed year of transmission, as others have done [30, 44, 45]. However, it has been reported that the interval between onset of drug injection and HCV infection has lengthened in recent years [46], which may indicate that the duration of infection could be shorter than estimated in our model of fibrosis progression. If so, our fibrosis model produces a spuriously slow rate of fibrosis progression, making the prognosis relatively conservative.

Sixth, the Markov model assumes that HCC only occurs when liver cirrhosis is established (Ishak 6). However, HCC can develop in lower fibrosis stages in chronic HCV infection [47, 48]. Finally, regression of fibrosis, cirrhosis and cirrhosis complications is possible after achieving SVR [49–51]. We do not have data to assess the effect of SVR on fibrosis regression, i.e. available pretreatment and post-treatment liver biopsies, and this is another limitation of the model. However, removing subjects achieving SVR from the model can mimic fibrosis regression in non-cirrhotic cases. In patients with cirrhosis, about 60% can regress after SVR [49, 50], and much less likely in decompensated cirrhosis. In patients with HCV-induced cirrhosis who attain SVR, the risk of HCC declines, but persists [52, 53]. To reflect that model cases with cirrhosis and SVR still are in risk of cirrhosis complications, we have retained 50% of these in the model.

#### Implications

HCV is the leading cause of chronic liver disease and cirrhosis and is the main cause of liver transplantation in the Western world [10]. Although the total number of HCV-infected individuals is estimated to be stable or decline in the future, an increase in liver cirrhosis, liver cancer, hepatic decompensation and liver-related deaths is expected in the coming years.

This assessment is underlined by the World Health Organization (WHO) statement that the burden of HCV disease has been largely ignored as a health priority, and the organization has developed the first-ever global health sector strategy for addressing the viral hepatitis pandemic [54].

To reduce the future burden of hepatitis C it is necessary to meet the challenge at three levels. First, prophylaxis measures must focus on reducing the transmission rate among active injection drug users. In addition, recent guidelines suggest that treatment should be offered to this group of individuals at high risk of transmitting HCV due to a potential prevention benefit [55]. Second, HCV infection among apparently healthy subjects must be diagnosed by screening of high-risk groups. Strategies to expand screening beyond high-risk groups should be considered since a substantial proportion of infected are unaware of their status [56]. Third, antiviral treatment has been offered only to a small percentage of patients, as treatment is hampered by the high cost of the new direct-acting antiviral drugs (DAAs). Based on the unpredictable course of liver fibrosis at the individual level, delaying treatment of patients with early fibrosis stages will increase the risk of liver complications [11]. DAAs has the potential to reduce the future burden of disease of HCV, but this is restricted by the current treatment levels [19]. In spite of the high costs of DAAs, several studies show that interferon-free regimens are cost-effective compared to interferon-based regimens [57–61].

## Conclusion

Based on the registration of patients with HCV in a low risk area, we estimate a relatively slow fibrosis progression within the first 20–25 years of infection, followed by an accelerated fibrosis progression, especially in subjects with HCV genotype 3. This may have important implications in the clinical management of patients infected with genotype 3.

Furthermore, we estimate a gradual increase in future complications with an estimated peak around 2040. The projected scenario implies a substantial increase in HCV-related morbidity and mortality in the coming years. An increased number of patients need to be treated to have an impact on the future burden of HCV disease.

## Additional files

Additional file 1: Ordinal regression model for estimating fibrosis grade according to duration of infection. (DOCX 19 kb)

Additional file 2: Estimated HCV cohort 1992–2012. The file describes how we estimated our HCV cohort. (DOCX 15 kb)

## Abbreviations

DAA: Direct acting antiviral
HCC: Hepatocellular carcinoma
HCV: Hepatitis C virus
SVR: Sustained virological response
WHO: World Health Organization
