# Supplementary material for: Future complications of chronic hepatitis C in a low-risk area: projections from the hepatitis c study in Northern Norway
Source: BMC Infect Dis. 2017 Sep 16;17:624. doi: 10.1186/s12879-017-2722-0 (PMC5602833; doi:10.1186/s12879-017-2722-0)
Supplement: Supplementary file 1 — Ordinal regression model for estimating fibrosis grade according to duration of infection. (DOCX 19 kb) [file 12879_2017_2722_MOESM1_ESM.docx]

Ordinal regression model for estimating fibrosis grade according to duration of infection.

Dataset of 237 records with known time of transmission and a liver biopsy available from the time of diagnosis. This sample has the same demographic characteristics as the main cohort regarding genotype, sex and age.

The analysis is performed in SPSS.

| **Case Processing Summary** | | | |
| --- | --- | --- | --- |
|  | | N | Marginal Percentage |
| Fibrosis (Ishak) | 0 | 26 | 11,0% |
|  | 1 | 48 | 20,3% |
|  | 2 | 84 | 35,4% |
|  | 3 | 33 | 13,9% |
|  | 4 | 11 | 4,6% |
|  | 5 | 14 | 5,9% |
|  | 6 | 21 | 8,9% |
| Genotype | 1 or 4 | 110 | 46,4% |
|  | 2 | 23 | 9,7% |
|  | 3 | 104 | 43,9% |
| Valid | | 237 | 100,0% |
| Missing | | 0 |  |
| Total | | 237 |  |

| **Model Fitting Information** | | | | |
| --- | --- | --- | --- | --- |
| Model | -2 Log Likelihood | Chi-Square | df | Sig. |
| Intercept Only | 568,497 |  |  |  |
| Final | 526,249 | 42,248 | 3 | ,000 |
| Link function: Logit. | | | | |

| **Pseudo R-Square** | |
| --- | --- |
| Cox and Snell | ,163 |
| Nagelkerke | ,169 |
| McFadden | ,051 |
| Link function: Logit. | |

| **Parameter Estimates** | | | | | | | | |
| --- | --- | --- | --- | --- | --- | --- | --- | --- |
|  | | Estimate | Std. Error | Wald | df | Sig. | 95% Confidence Interval | |
|  |  |  |  |  |  |  | Lower Bound | Upper Bound |
| Threshold | [Ishak = 0] | -1,088 | ,260 | 17,547 | 1 | ,000 | -1,597 | -,579 |
|  | [Ishak = 1] | ,329 | ,225 | 2,133 | 1 | ,144 | -,113 | ,771 |
|  | [Ishak = 2] | 2,016 | ,262 | 59,232 | 1 | ,000 | 1,502 | 2,529 |
|  | [Ishak = 3] | 2,850 | ,295 | 93,555 | 1 | ,000 | 2,273 | 3,428 |
|  | [Ishak = 4] | 3,218 | ,312 | 106,160 | 1 | ,000 | 2,606 | 3,830 |
|  | [Ishak = 5] | 3,840 | ,350 | 120,676 | 1 | ,000 | 3,155 | 4,525 |
| Location | [Gen_sim=1] * Duration | ,074 | ,016 | 21,559 | 1 | ,000 | ,043 | ,105 |
|  | [Gen_sim=2] * Duration | ,085 | ,021 | 15,787 | 1 | ,000 | ,043 | ,126 |
|  | [Gen_sim=3] * Duration | ,094 | ,016 | 36,574 | 1 | ,000 | ,064 | ,125 |
| Link function: Logit. | | | | | | | | |

| **Test of Parallel Lines^a^** | | | | |
| --- | --- | --- | --- | --- |
| Model | -2 Log Likelihood | Chi-Square | df | Sig. |
| Null Hypothesis | 526,249 |  |  |  |
| General | 506,751^b^ | 19,498^c^ | 15 | ,192 |
| The null hypothesis states that the location parameters (slope coefficients) are the same across response categories. | | | | |
| a. Link function: Logit. | | | | |
| b. The log-likelihood value cannot be further increased after maximum number of step-halving. | | | | |
| c. The Chi-Square statistic is computed based on the log-likelihood value of the last iteration of the general model. Validity of the test is uncertain. | | | | |

From these estimates the value of the logit function can be calculated for each threshold, genotype, and year of transmission by the formula: $logit=threshold-duration\times location$. This matrix of logit values can then be transformed to a probability distribution for each genotype, fibrosis grade and year of duration using the formula: $Cumulated probability=\frac{e^{logit}}{1+e^{logit}}$

From the matrix of cumulated probability a matrix of probability distributions over the fibrosis scale can be made for each genotype and year of duration.

The model predicted 72% of the fibrosis states correctly within +/- one fibrosis grade. Overestimated in 12 % and underestimated in 16%.

|  |  | Fibrosis (Ishak) | |  |  |  |  |  |
| --- | --- | --- | --- | --- | --- | --- | --- | --- |
|  |  | Observed fibrosis category | | |  |  |  |  |
|  |  | 0 | 1 | 2 | 3 | 4 | 5 | 6 |
| Predicted fibrosis | | Count | Count | Count | Count | Count | Count | Count |
| category | 0 | 0 | 0 | 0 | 0 | 0 | 0 | 0 |
|  | 1 | 4 | 7 | 0 | 1 | 0 | 0 | 1 |
|  | 2 | 22 | 41 | 80 | 31 | 9 | 13 | 14 |
|  | 3 | 0 | 0 | 0 | 0 | 0 | 0 | 0 |
|  | 4 | 0 | 0 | 0 | 0 | 0 | 0 | 0 |
|  | 5 | 0 | 0 | 0 | 0 | 0 | 0 | 0 |
|  | 6 | 0 | 0 | 4 | 1 | 2 | 1 | 6 |
|  |  | Total: |  | 237 | 100 % |  |  |  |
|  |  | Good |  | 170 | 72 % |  |  |  |
|  |  | Bad |  | 67 | 28 % |  |  |  |
|  |  | Overestimated | | 29 | 12 % |  |  |  |
|  |  | Underestimated | | 38 | 16 % |  |  |  |
