# Supplementary material for: Future complications of chronic hepatitis C in a low-risk area: projections from the hepatitis c study in Northern Norway
Source: BMC Infect Dis. 2017 Sep 16;17:624. doi: 10.1186/s12879-017-2722-0 (PMC5602833; doi:10.1186/s12879-017-2722-0)
Supplement: Supplementary file 2 — Estimated HCV cohort 1992–2012. The file describes how we estimated our HCV cohort. (DOCX 15 kb) [file 12879_2017_2722_MOESM2_ESM.docx]

**Estimated HCV cohort 1992 -2012**

*The year of diagnosis* was defined as the first year of a positive anti-HCV test. Until 2004, a positive anti-HCV test was directly confirmed with a recombinant immunoblot assay (RIBA). The HCV-RNA test replaced the RIBA test for confirmation of HCV infection from 2005.

The registration of HCV infection revealed *incomplete records* regarding confirmation testing. Not all the persons with positive anti-HCV tests had a recorded confirmation test. The starting cohort in the model thus consists of patients with confirmed HCV infection (either a positive RIBA or a positive HCV RNA test), as well as individuals with unconfirmed HCV (either only a positive anti-HCV or an indeterminate RIBA). We estimated the likelihood of a true positive test in incomplete records in the following way:

In a sample of 326 records with a positive anti-HCV test where RIBA had been measured, 207 subjects (63%) had a positive RIBA test, and the probability of a true positive anti-HCV test was estimated to 0.63. Similarly, in a sample of 14 records with an inconclusive RIBA and a HCV RNA test, we found three individuals with a positive HCV RNA test, and the probability of a true positive record in case of inconclusive RIBA was estimated to 0.21.

Summarized, individuals with either a positive HCV RNA or RIBA were weighted *1.0*, and individuals with only a positive anti-HCV test or an inconclusive RIBA were weighted *0.63* and *0.21*, respectively.

**HCV cohort** **1992-1997** (*Kristiansen et.al 2010*):

1992: 66

1993: 119

1994: 130

1995: 95

1996: 119

1997: 127

Total: **656** (all confirmed by either a positive HCV RNA test or a positive RIBA test)

**HCV cohort 1998-2012:**

| **Year** | Positive HCV-RNA | Positive RIBA ( | Only positive anti-HCV | Positive anti-HCV and indeterminate RIBA | **Total** |
| --- | --- | --- | --- | --- | --- |
| **1998** | 88 | 60 | 1 | 3 | 152 |
| **1999** | 115 | 65 | 0 | 2 | 182 |
| **2000** | 91 | 54 | 1 | 1 | 147 |
| **2001** | 81 | 48 | 0 | 3 | 132 |
| **2002** | 130 | 38 | 0 | 4 | 172 |
| **2003** | 120 | 78 | 0 | 8 | 206 |
| **2004** | 124 | 54 | 2 | 6 | 186 |
| **2005** | 66 | 7 | 24 | 1 | 98 |
| **2006** | 66 | 7 | 18 | 2 | 93 |
| **2007** | 67 | 6 | 23 | 2 | 98 |
| **2008** | 62 | 17 | 14 | 2 | 95 |
| **2009** | 59 | 10 | 22 | 3 | 94 |
| **2010** | 63 | 14 | 20 | 2 | 99 |
| **2011** | 65 | 11 | 14 | 1 | 91 |
| **2012** | 64 | 13 | 10 | 1 | 88 |
|  | 1261 | 482 | 149 | 41 | **1933** |

**Estimated HCV cohort 1992 -2012:** 656 + 1933 = **2589**
